# Long-Term Biodistribution and Safety of Human Dystrophin Expressing Chimeric Cell Therapy After Systemic-Intraosseous Administration to Duchenne Muscular Dystrophy Model

**DOI:** 10.1007/s00005-022-00656-7

**Published:** 2022-08-17

**Authors:** Maria Siemionow, Sonia Brodowska, Paulina Langa, Kristina Zalants, Katarzyna Kozlowska, Wictoria Grau-Kazmierczak, Ahlke Heydemann

**Affiliations:** 1 Poznan University of Medical Sciences, Poznan, Poland; 2 Department of Orthopaedics, University of Illinois at Chicago, Molecular Biology Research Building, 900 S. Ashland Ave. Room# 3356, Chicago, IL 60607 USA; 3 Department of Physiology and Biophysics, University of Illinois at Chicago, Chicago, IL USA

**Keywords:** Duchenne muscular dystrophy, Dystrophin expressing chimeric cells, Systemic-intraosseous administration, Therapy safety, Biodistribution, Advanced therapy medicinal product

## Abstract

Duchenne muscular dystrophy (DMD) is a lethal disease caused by X-linked mutations in the dystrophin gene. Dystrophin deficiency results in progressive degeneration of cardiac, respiratory and skeletal muscles leading to premature death due to cardiopulmonary complications. Currently, no cure exists for DMD. Based on our previous reports confirming a protective effect of human dystrophin expressing chimeric (DEC) cell therapy on cardiac, respiratory, and skeletal muscle function after intraosseous administration, now we assessed long-term safety and biodistribution of human DEC therapy for potential clinical applications in DMD patients. Safety of different DEC doses (1 × 10^6^ and 5 × 10^6^) was assessed at 180 days after systemic-intraosseous administration to *mdx/scid* mice, a model of DMD. Assessments included: single cell gel electrophoresis assay (COMET assay) to confirm lack of genetic toxicology, magnetic resonance imaging (MRI) for tumorigenicity, and body, muscle and organ weights. Human DEC biodistribution to the target (heart, diaphragm, gastrocnemius muscle) and non-target (blood, bone marrow, lung, liver, spleen) organs was detected by flow cytometry assessment of HLA-ABC markers. Human origin of dystrophin was verified by co-localization of dystrophin and human spectrin by immunofluorescence. No complications were observed after intraosseous transplant of human DEC. COMET assay of donors and fused DEC cells confirmed lack of DNA damage. Biodistribution analysis of HLA-ABC expression revealed dose-dependent presence of human DEC cells in target organs, whereas negligible presence was detected in non-target organs. Human origin of dystrophin in the heart, diaphragm and gastrocnemius muscle was confirmed by co-localization of dystrophin expression with human spectrin. MRI revealed no evidence of tumor formation. Body mass and muscle and organ weights were stable and comparable to vehicle controls, further confirming DEC safety at 180 days post- transplant. This preclinical study confirmed long-term local and systemic safety of human DEC therapy at 180 days after intraosseous administration. Thus, DEC can be considered as a novel myoblast based advanced therapy medicinal product for DMD patients.

## Introduction

Duchenne muscular dystrophy (DMD) is a lethal X-linked recessive genetic disease, characterized by mutations of the dystrophin gene leading to fibrosis, inflammation and progressive weakness and degeneration of cardiac, respiratory and skeletal muscles resulting in premature death of DMD patients.

Currently, there is no cure for DMD and the recommended supportive therapies include corticosteroid and physical therapy with a focus on amelioration of symptoms and improvement of quality of life of DMD patients (Falzarano et al. 2015).

Thus, there is a significant scientific effort to search for novel therapeutic approaches which will be both efficacious and safe. There are two major interests in DMD therapeutics. The first, aims to restore dystrophin expression and function. The second, targets the pathological changes associated with DMD (Guiraud and Davies 2017). In this respect, there are numerous preclinical and clinical approaches for DMD restoration: stem cell based therapies (Barthélémy and Wein 2018; Biressi et al. 2020; Sienkiewicz et al. 2015), CRISPR-mediated gene editing (Min et al. 2019; Nelson et al. 2016), exon skipping (Barthélémy and Wein 2018; Kinali et al. 2009), and gene replacement via recombinant adeno-associated virus (AAV)-based vectors (Barthélémy and Wein 2018).

The long-term clinical efficacy of these therapies has been confirmed in recent reports (Nelson et al. 2019; Servais et al. 2022; Xu et al. 2019), however the risks associated with off-target mutations and tumorigenicity still raise significant safety concerns.

Safety-related problems were reported both in mice and the non-human primates for the antisense oligonucleotides-mediated exon skipping therapy for DMD (Gait et al. 2019; Sun et al. 2020). Furthermore, analysis of the vector-mediated gene therapy tested in the golden retriever dog model demonstrated a concerning immune response of inflammatory myopathy and progressive liver dysfunction associated with a high dose of AAVs (Kornegay et al. 2010). It is clear that many of the DMD therapies under development share one common concern: the therapy safety, thus the major scientific efforts focus on the reduction of drug toxicity while maintaining the therapeutic efficacy.

In contrast to gene therapies, several cell-based therapies tested in DMD patients were successful both in terms of safety and efficacy. Specifically, allogenic myoblast transplantation in a clinical scenario was confirmed to be safe as reported by Gussoni et al. (1997) and Skuk and Tremblay (2015). Moreover, more recent studies testing cell-based DMD therapies confirmed safety of transplantation of autologous bone marrow-derived mononuclear cells (Sharma et al. 2013) and human umbilical cord blood-derived mesenchymal stem cells (MSCs) (Rajput et al. 2015).

However, despite the proven safety of the myoblast transplantation, the efficacy of cell engraftment and the need for immunosuppression to prevent cell rejection, represents the outstanding concern preventing the routine clinical application of the cell-based therapies in DMD.

To address these concerns, we have previously proposed and reported application of myoblast therapy for DMD based on our well-established cell fusion technology (Siemionow et al. 2018a, 2018b), and created a new line of human dystrophin expressing chimeric (DEC) cells via ex-vivo fusion of human myoblasts from normal and DMD-affected donors. In vitro, DEC displayed phenotype and genotype of the parent cells, expressed dystrophin and maintained proliferative and myogenic differentiation potential (Siemionow et al. 2018b). Moreover, tolerogenic and immunomodulatory properties of DEC were confirmed in our previous report (Siemionow et al. 2021b).

We have further tested effect of DEC in the immunocompetent mdx mice model and confirmed significant increase in dystrophin expression which correlated with improvement of muscle function after both, the local-intramuscular and the systemic-intraosseous administration of DEC cells created from two unrelated donors, where there was no evidence of adverse side effects and no need for immunosuppression (Siemionow et al. 2018a, 2019). In addition, we have also confirmed in the mdx and *mdx/scid* mouse models of DMD, that systemic-intraosseous DEC administration resulted in the long-term amelioration of the cardiac, pulmonary and skeletal muscle function which correlated with increased dystrophin expression, improved muscle morphology and reduced mdx pathology (Siemionow et al. 2019, 2021a, 2022).

Therefore, considering recent reports on the increased drug toxicity observed in gene therapies, as well as to address the current limitations of the allogeneic myoblast-based therapies, the primary goal of the current study was to evaluate the long-term safety of DEC cells at 180 days after systemic-intraosseous administration, in preparation for clinical application of DEC therapy in DMD patients. Furthermore, we have previously reported DEC efficacy, confirming a significant increase in dystrophin expression which correlated with improvement of function in the cardiac, respiratory and skeletal muscles (Siemionow et al. 2018a, 2018b, 2019, 2021a, 2022).

In this study we first addressed safety of the intraosseous DEC injection, evaluating both the local and systemic effects. Next, as an important safety measure, we checked DEC cell engraftment and biodistribution to target and non-target organs, as well as investigated any potential tumor formation by weekly palpation, by magnetic resonance imaging (MRI), and at the autopsy. The effect of DEC therapy on the animal’s well-being was assessed weekly by experienced veterinarians and body and organ weights were taken to further assess DEC therapy safety.

In summary, in this study we confirmed long-term safety of dystrophin expressing chimeric cell therapy as confirmed by the lack of DNA damage after DEC fusion, lack of local toxicity at the DEC injection site, lack of tumor formation by MRI and at the autopsy, biodistribution of DEC to the selected target organs and negligible presence in the non-target organs. Moreover, the muscle and organ weights were comparable with the vehicle-injected controls, further confirming DEC safety. This encouraging safety data introduces DEC as a novel therapeutic modality of advanced therapy medicinal product (ATMP) to the armamentarium of therapeutic options for DMD patients.

## Materials and Methods

### Mice and Animal Care

This study was approved by the Institutional Animal Care and Use Committee (IACUC) of University of Illinois at Chicago, which is approved by the American Association for the Accreditation of Laboratory Animal Care (AAALAC). All animals received humane care in compliance with the “Principles of Laboratory Animal Care” formulated by the National Society for Medical Research and the “Guide for the Care and Use of Laboratory Animal Resources”. Six- to 8 week-old male *mdx/scid* mice—the animal model for Duchenne muscular dystrophy (B10ScSn.Cg-Prkdcscid Dmdmdx/J, stock number 018018) were purchased from Jackson Laboratories. Animals were kept in a pathogen-free environment on a light/dark cycle. Prior to study initiation, aged matched male *mdx/scid* mice were ear tagged and randomized into experimental groups: vehicle-injected animals with 60 µl phosphate-buffered saline—PBS (*n* = 4), animals injected with 1 × 10^6^ DEC cells suspended in 60 µl PBS (*n* = 5), and animals injected with 5 × 10^6^ DEC cells suspended in 60 µl PBS (*n* = 5). All treated animals were observed daily after injection of the DEC cells or saline for any local signs of infection, edema or hematoma or animal distress for the first week. No signs of local changes were observed, and treatment had no effect on general animal conditions, food consumption and coat changes. Animal observations were carried out up to 180 days after administration of DEC therapy and there were no acute or delayed signs of immune response (anaphylactic reaction, edema, erythema, inflammatory response) observed.

### Creation of the Human DEC Therapy

#### Cell Culture

Normal human myoblasts were purchased from Lonza Bioscience (Mapleton, IL, USA), and DMD-affected myoblasts were purchased from Creative Bioarray Ltd. (Little Chesterford, UK). Myoblasts (MB) were cultured in Skeletal Muscle Cell Growth Medium-2 (Lonza Clonetics, Mapleton, IL, USA) supplemented with: human epidermal growth factor; fetal bovine serum (FBS); Dexamethasone; Gentamicin/Amphotericin B from Lonza Clonetics. Upon reaching 60–70% confluence, myoblasts were harvested using 0.25% trypsin/EDTA (Sigma-Aldrich, MO, USA). Enzymatic activity was inhibited with 10% serum-supplemented culture media. Human MBs were harvested between passages 3–7, which is optimal for an ex vivo cell fusion procedure.

#### Cell Fusion

After harvesting, cell viability was assessed with 0.4% Trypan Blue staining (Gibco- ThermoFischer, Waltham, MA, USA), parent myoblasts (MB^N^ and MB^DMD^) were washed in serum-free media supplemented with antibiotics (1% Antibiotic–Antimycotic solution, Gibco-ThermoFischer, Waltham, MA, USA). Then, our standard cell fusion procedure was performed. As previously described (Siemionow et al. 2018a, 2018b, 2021a) parent myoblasts (MB^N^ and MB^DMD^) were fluorescently labeled using PKH26 or PKH67 (Sigma-Aldrich, St. Louis, MO, USA) membrane dyes according to the manufacturer’s instructions. Parent cells were mixed and washed, then fusion was performed using a 1.46 g/mL PEG solution (PEG 4000, EMD) containing 16% DMSO (Sigma, St. Louis, MO, USA).

#### Cell Sorting Procedure

Fused cells were transferred to the fluorescently activated cells sorting (FACS) buffers containing 5% HEPES, 1% EDTA and 5% FBS. Cells presenting double positive (PKH26/PKH67) staining were selected via FACS (MoFlow Astrios, Beckman Coulter, San Jose, CA, USA) and used for in vitro analysis in preparation for transplantation to *mdx/scid* mice. The cells were counted and examined for viability, cluster differentiation (CD) markers and staining efficacy. After sorting, DEC cells were suspended in 10% serum-supplemented with culture media. DEC cells were further propagated in Lonza Clonetics supplemented with FBS to achieve an optimal number of cells for transplantation.

#### Preparation of Human DEC Cell Suspension

DEC cells were harvested between passages 3 and 7 after cell fusion, which is the optimal number of passages to receive a sufficient number of cells for intraosseous administration to the recipient mice. Next, DEC cells were counted and viability of cells was evaluated, then two doses of 1 × 10^6^ and 5 × 10^6^ of DEC cells were suspended in 60 μl of PBS and transferred into the tuberculin syringe with 25G needle (Becton, NJ, USA). The same volume of 60 μl of PBS without DEC cells was injected into the vehicle control group.

### Systemic–Intraosseous DEC Transplantation

Mice were anesthetized with 2% isoflurane inhalation along with 1 ml/kg buprenorphine subcutaneous injection. DEC intraosseous transplant was performed as previously reported (Siemionow et al. 2019). Briefly, a 5 mm incision was made in the lateral-mid right thigh and muscles were separated to expose the femoral bone. DECs were transferred in a 60 µl volume of sterile PBS to a tuberculin syringe (ThermoFischer, Waltham, MA, USA). A 25G needle was used to aspirate 60 µl of femoral bone marrow followed by DEC cells injection directly into the femur. Bone wax (Medline Industries, Inc, Mundelein, IL, USA) was applied to the injection site, the muscles were re-approximated, and the wound was closed with 5–0 nylon sutures (Ethicon, LLC, Mexico). Animals recovered in a heated environment with post-operative monitoring and returned to the colony.

### Assessment of Safety of Human DEC Therapy

#### Assessment of Cell Fusion Safety by Single Cell Gel Electrophoresis COMET Assay

To ensure safety of the therapy, an analysis was performed using the alkaline single cell gel electrophoresis according to the manufacturer (cat. STA-351–5/STA-351-5, Cell Biolabs, San Diego, CA, USA) instructions. Cells were resuspended at 1 × 10^6^ cells/mL in PBS and mixed with Comet Agarose at 1:10 ratio (v/v), and immediately layered onto microscope slides. Cells were assessed at different passages to confirm DNA stability and compared to the positive control with induced DNA-damage (cells treated with 100 μM hydrogen peroxide, H_2_O_2_). After standard single cell electrophoresis (300 mM NaOH, pH > 13, 1 mM EDTA), DNA was visualized using Vista Green dye diluted in TE buffer (1:10,000) and samples were examined immediately using LSM 710 fluorescence microscope Meta (Zeiss, Germany). Assessment of 50 cells randomly selected in images of therapy and control groups was performed by visual presence or lack of the “comet”-like tail and Comet Assay Software, CaspLab, to detect a “comet”-like tail which indicate the DNA damage.

#### Assessment of Local Safety and Tolerance

Following intraosseous DEC administration, the injection site was assessed daily for 2 weeks post-injection and on a monthly basis thereafter for any signs of bruising, redness, inflammation, infection or wound dehiscence. The analysis was performed by an experienced veterinarian from the College of Veterinary Medicine, University of Illinois at Chicago (USA).

### Assessment of Dose-Related Toxicity After Intraosseous Administration

To assess potential dose-dependent toxicity of DEC therapy, two different doses of 1 × 10^6^ and 5 × 10^6^ of human DEC cells suspended in 60 µl of saline and equivalent volume of saline in the control group, were injected via systemic intraosseous delivery route to the bone marrow compartment of the femoral bone. Animals were assessed during DEC therapy administration as well as for the following 72 h after DEC injection, for any signs of therapy related toxicity such as anaphylactic reaction, and local or systemic immune response. The analysis was performed by the experienced fellows and investigators from Siemionow Laboratory and by the experienced veterinarians from the College of Veterinary Medicine, University of Illinois at Chicago (USA).

### Assessment of Tumorigenicity

#### Assessment by Magnetic Resonance Scanning

In preparation for MRI imaging, mice were induced with 2% isoflurane, and then anesthesia was maintained with 1–1.5% in 100% oxygen throughout the experiment. A rectal probe was used to monitor body temperature, which was maintained at 37.5 °C by regulating the warm air flow into the scanner bore. Temperature and respiration rate were monitored using an MRI—compatible physiological monitoring system (Model 1025, SA Instruments Inc., Stony Brook, NY, USA). MRI was carried out by an Agilent Varian 9.4 T preclinical scanner with a 39-mm proton volume coil. Each mouse was scanned in three sections: brain, chest, and pelvis. T2 weighted images were acquired for each section and T1 for brain and liver (greater soft-tissue resolution facilitates tumor detection in solid organs). Scan parameters for T2 weighted images included: field of view 40 × 40 mm^2^, matrix size 128 × 128, TR = 2000 ms, echo train length 16, effective echo time 28 ms. Scan parameters for T1 included: field of view 40 × 40 mm^2^, matrix size 128 × 128, TR = 550 ms, echo time 28 ms. MRI scanning was performed before treatment and at day 30, 90 and 180 days after DEC transplantation.

#### Body and Organs Evaluation at Autopsy

Autopsy was performed at the study endpoint, at 180 days after systemic-intraosseous injection of DEC cells. The animals underwent euthanasia and were assessed by an experienced veterinarian for any signs of tumor or tumor-like formation, changes in organ size, texture or appearance, and presence of third-space body fluids.

#### Assessment of Body Mass and Muscle and Organs Weight

The baseline body mass of each animal was recorded at the beginning of the study and was then measured weekly after intraosseous DEC administration until the study endpoint at 180 days. In addition, at the study endpoint, the selected organs (heart, thymus, spleen, liver, lungs, kidney, spleen, and testis) and the skeletal muscles (diaphragm, right and left gastrocnemius, right and left tibialis anterior, right and left quadriceps, triceps, and right and left gluteus) were harvested after animal euthanasia, and dry weights of tissues of interest were recorded.

### Assessment of DEC Biodistribution to the Target and Non-target Organs

#### Flow Cytometry Analysis of the Human HLA-ABC Antigen for Confirmation of DEC Biodistribution and Engraftment

Evaluation of human DEC cells biodistribution included assessment of the presence of human HLA-ABC (human leukocyte antigen) marker in the target organs (heart, diaphragm, gastrocnemius muscle) and non-target organs (liver, spleen, lung, kidney, and bone marrow extracted from right femoral bone cavity—DEC injection site) analyzed by flow cytometry. Biodistribution of human DEC cells to the organs was evaluated by staining of the cell solution isolated from the respective organs with the anti-human mouse derived HLA-ABC antibody conjugated with allophycocyanin (APC) (cat. 55,555, BD Biosciences) for 40 min, in the dark, at 4 °C. Next, cells were washed with PBS supplemented with 1% BSA, then fixed using 4% paraformaldehyde, washed with PBS and assessed using flow cytometry (Gallios, Beckman Coulter, San Diego, CA, USA) detecting the APC signal. Biodistribution studies were performed at the study endpoint of 180 days after DEC transplant, to assess cell migration and persistence of human DEC cells within the selected target organs.

#### Immunofluorescence Analysis for Confirmation of Human Origin of Dystrophin by Co-localization of Dystrophin with the Human Spectrin

To assess the migration and check the presence of human DEC cells after 180 days, the staining for dystrophin and human spectrin was performed on optimal cutting temperature frozen sections of the heart, diaphragm and gastrocnemius muscle (GM) as described previously (Siemionow et al. 2021a) Specimens were incubated with rabbit—polyclonal anti-human dystrophin antibody (1:50, cat. ab15277, Abcam, Cambridge, UK) and mouse monoclonal anti-human spectrin (Leica, Clone RBC2/3D5, Biosystems, NCL-SPEC1) primary antibodies, followed by incubation with a goat-anti rabbit Alexa Fluor 488-conjugated secondary antibody (1:400, A-21241, ThermoFischer, Waltham, MA, USA) and goat anti-mouse conjugated AlexaFluor-647 secondary antibody for dystrophin (1:400, ab150115, Abcam, Cambridge, UK). Appropriate positive and negative controls were used. Nuclear counter-staining was performed using 4ʹ,6-diamidino-2-phenylindole (DAPI) (cat. ab104139, Abcam, Cambridge, UK). A Zeiss Meta confocal microscope with ZEN software (Carl Zeiss, Oberkochen, Germany) and Leica DM4000B microscope were used for fluorescence signal detection and analysis.

### Statistical Analysis

Data are expressed as mean ± SEM (standard error of the mean). GraphPad Prism (ver 9.2.1) software was used to perform statistical analysis. Two-tailed Student *t*-test for group comparisons and Two-way ANOVA with Sidak’s multiple comparisons test were used to define statistical significance. Results were considered statistically significant for *p* < 0.05.

## Results

### COMET Assay Confirmed Safety of Human DEC Therapy for Systemic-Intraosseous Administration to the *mdx/scid* Mice

**Fig. 1:**
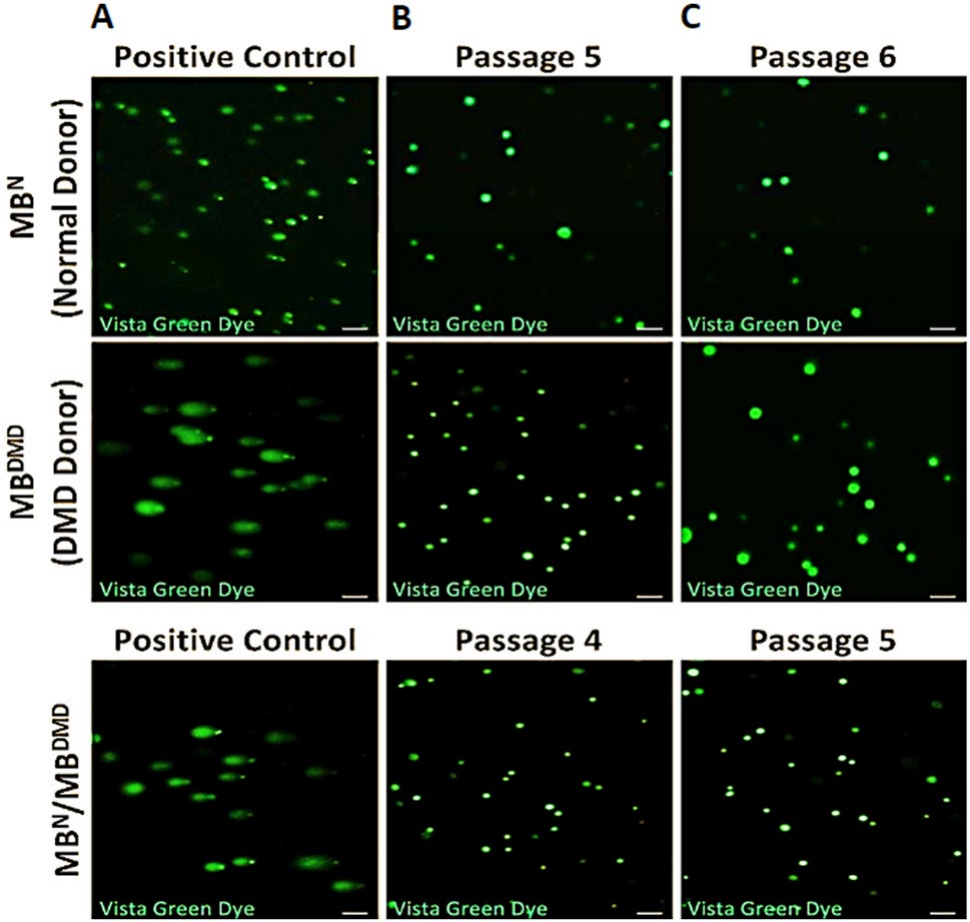
Single cell gel electrophoresis (COMET) assay confirms safety and lack of genotoxicity of the ex vivo cell fusion protocol applied for the creation of the MB^N^/MB^DMD^ DEC therapy for intraosseous administration to the *mdx/scid* mice. **A.** Representative images of the COMET assay of the positive control cells treated with 100 μM H_2_O_2_ (left vertical column). **B.** Confirmation of DNA stability of normal parent cells (MB^N^) prior to cell fusion, and DMD-affected parent cells (MB^DMD^) prior to cell fusion at passage 5, and DEC cells (MB^N^/MB^DMD^) created by fusion of the parent cells at passage 4 (lower panel) (middle vertical column). **C.** Confirmation of DNA stability of normal parent cells prior to fusion, DMD-affected parent cells prior to fusion at passage 6, and MB^N^/MB^DMD^ DEC cells at passage 5 (right vertical column). Images were captured using LSM 710 fluorescence microscope Meta (Zeiss, Germany), Magnification 5 × , scale bars 100 μm

COMET Assay performed for detection of the possible DNA damage in the in vitro propagated and passaged (P6) parent cells (MB^N^ and MB^DMD^) as well as passaged (P5) DEC (MB^N^ /MB^DMD^) cells, revealed lack of DNA damage confirming lack of genotoxicity in cell culture up to passage 6 as confirmed by the lack of a “comet like tail” structures in the cultured DEC cells, whereas the “comets'' were observed in the positive control cells treated with hydrogen peroxide to induce DNA damage. These results confirmed DNA stability and safety of the cell fusion procedure, and safety of in vitro cells propagation before intraosseous administration of DEC to the *mdx/scid* mouse model (Fig. 1).

### Long-term Local Safety and Tolerance was Confirmed at 180 days After Intraosseous Administration of Human DEC Therapy

Following intraosseous DEC transplantation, animals were observed weekly by board-certified veterinarians from the Veterinary Diagnostic Laboratory at the College of Veterinary Medicine, at the University of Illinois at Chicago (USA). Specifically, the injection site was assessed on a daily basis for 2 weeks post-transplantation and at a monthly basis thereafter and there were no signs of bruising, redness, inflammation, infection or wound dehiscence observed at the injection site or reported in the animal’s individual charts over the entire 180 day observation period. During routine animal control, one mouse developed a mass on the right hind limb at 90 days after DEC (1 × 10^6^) administration and was euthanized before the study endpoint. The pathology assessment revealed sarcoma of mouse origin which is prevalent in the *mdx/scid* mice prone to tumor formation (Santagostino et al. 2017).

### Lack of Dose-Related Toxicity was Confirmed After Intraosseous Administration of Human DEC Therapy

Assessment of animals during intraosseous administration of the lower DEC dose of 1 × 10^6^ as well as during injection of 5 × higher dose of 5 × 10^6^, revealed no side effects during therapy administration nor dose-related toxicity after administration of the higher DEC dose. Moreover, close monitoring of animals for 72 h after administration of both DEC doses did not reveal any therapy related toxicity such as anaphylactic reaction, local or systemic immune response, thus confirming safety of DEC therapy. Furthermore, based on the weekly veterinarian observations and palpation there were no reports on the presence of organ enlargement or fluid accumulation during the entire 180 days of evaluation. Moreover, at the study end-point all animals were euthanized and subjected to the full organ necropsy (including liver and kidney weight measurements and macroscopic examination) performed by the experienced veterinarian and study investigators. There were no signs of organ enlargement or shrinkage, no signs of inflammation or presence of fluid within the abdominal cavity. In addition, the organs of animals treated with both doses of DEC therapy were harvested at the necropsy, weighed and compared with the organs of the control—saline injected animals and there were no abnormalities found in the organs size, appearance, texture or weight at 180 days after DEC administration confirming lack of toxicity after systemic-intraosseous administration of two different doses of DEC therapy.

### Human DEC Therapy Safety was Confirmed by Magnetic Resonance Scanning and Autopsy Assessment, Revealing Lack of Tumor Formation up to 180 days After Systemic-Intraosseous Administration of Human DEC Therapy

**Fig. 2:**
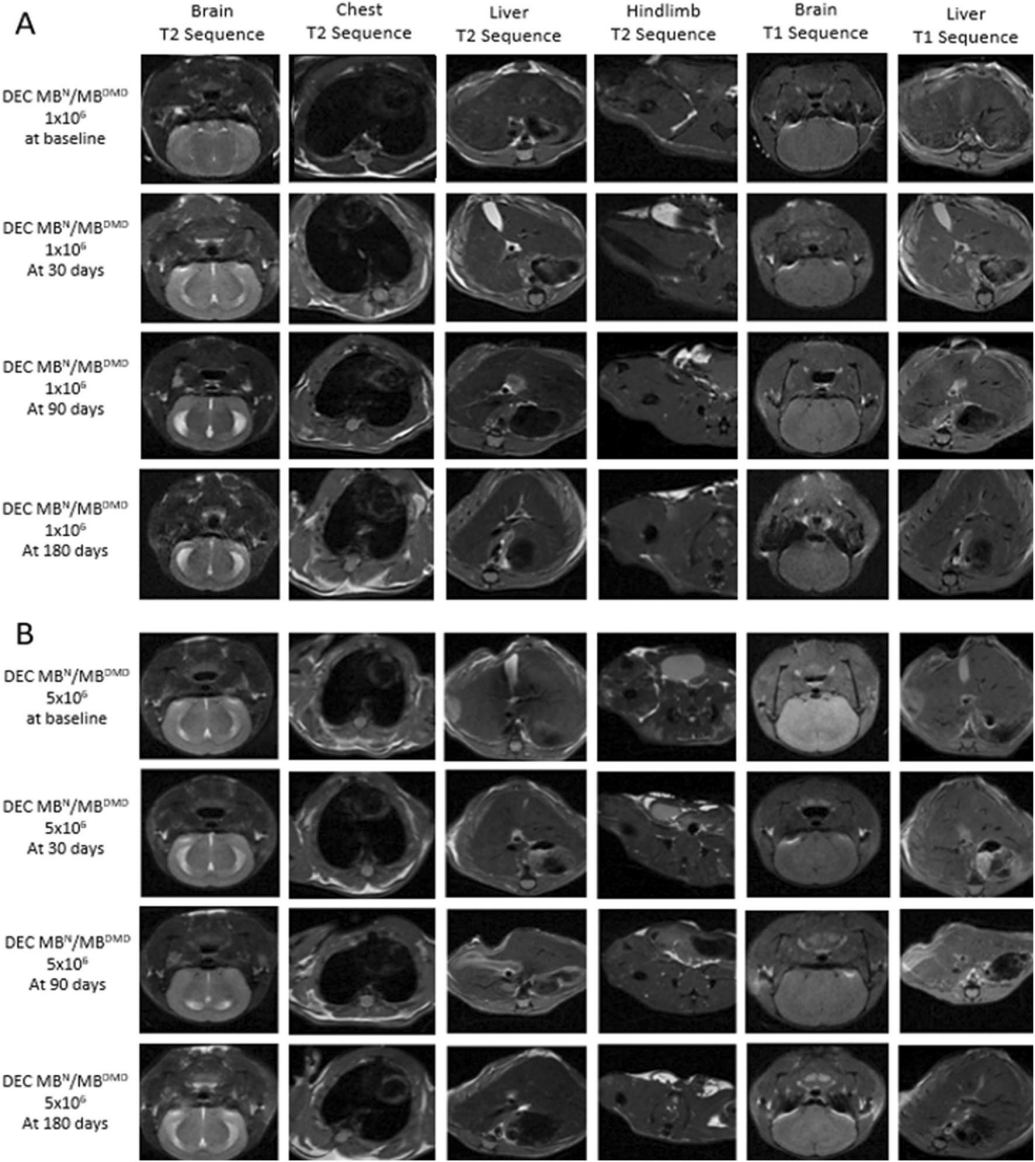
Magnetic Resonance Imaging (MRI) confirms the lack of tumor formation and safety of human Dystrophin Expressing Chimeric (DEC) Therapy assessed up to 180 days after intraosseous administration of two doses (1 × 10^6^ and 5 × 10^6^) of DEC Therapy. Safety assessment of DEC therapy by comparative analysis of MRI images at time 0 before DEC injection and at 30, 90, and 180 days after intraosseous administration of **A.** 1 × 10^6^ DEC and **B.** 5 × 10^6^ DEC. Evaluation of organs at the highest tumor risk rate (brain, chest, liver) and the right hind-limb (the femoral shaft level of the DEC injected limb) confirmed lack of tumor formation in all tested organs up to day 180 post-DEC administration (31 cm bore size 9.4 Tesla Agilent MRI System) (Model 1025, SA Instruments Inc., Stony Brook, NY, USA). Each mouse was scanned at each time-point at three sections: brain, chest, and pelvis. T2 weighted images were acquired for each section and T1 weighted images were acquired for brain and liver (greater soft-tissue resolution facilitates tumor detection in solid organs). Scan parameters for T2 weighted images: field of view 40 × 40mm^2^, matrix size 128 × 128, TR = 2000 ms, echo train length 16, effective echo time 28 ms. Scan parameters for T1 weighted images: field of view 40 × 40mm^2^, matrix size 128 × 128, TR = 550 ms, echo time 28 ms

Assessment of MRI images performed at 180 days, the study endpoint for both DEC therapy groups: the 1 × 10^6^ dose (Fig. 2A) and 5 × 10^6^ dose (Fig. 2B) and the vehicle-injected control, confirmed lack of tumor formation in the T2 weighted images acquired for each section of brain, chest and abdominal wall. Moreover, lack of tumor formation was confirmed on T1 weighted images acquired for brain and liver since greater soft-tissue resolution facilitates tumor detection in solid organs. These images acquired at 180 days study endpoint were comparable with the images taken before DEC administration and during the 30 and 90 day follow-up. These findings confirm lack of tumorigenicity and DEC therapy safety at long-term follow-up.

Autopsy assessment for the tumor formation at the study endpoint of 180 days was performed for all surviving animals from both DEC therapy groups by a trained veterinarian, and confirmed a lack of any signs of tumor or tumor-like structure formation. Moreover, macroscopic evaluation and organ weight measurements revealed no changes in the organ size, shape or texture, further confirming the safety and lack of toxicity of the systemic administration of two different doses of DEC therapy.

### Long-term Safety of Human DEC Therapy was Confirmed by Maintenance of Body Mass and Muscles and Organ Weights at 180 days After Systemic-Intraosseous DEC Administration

**Fig. 3:**
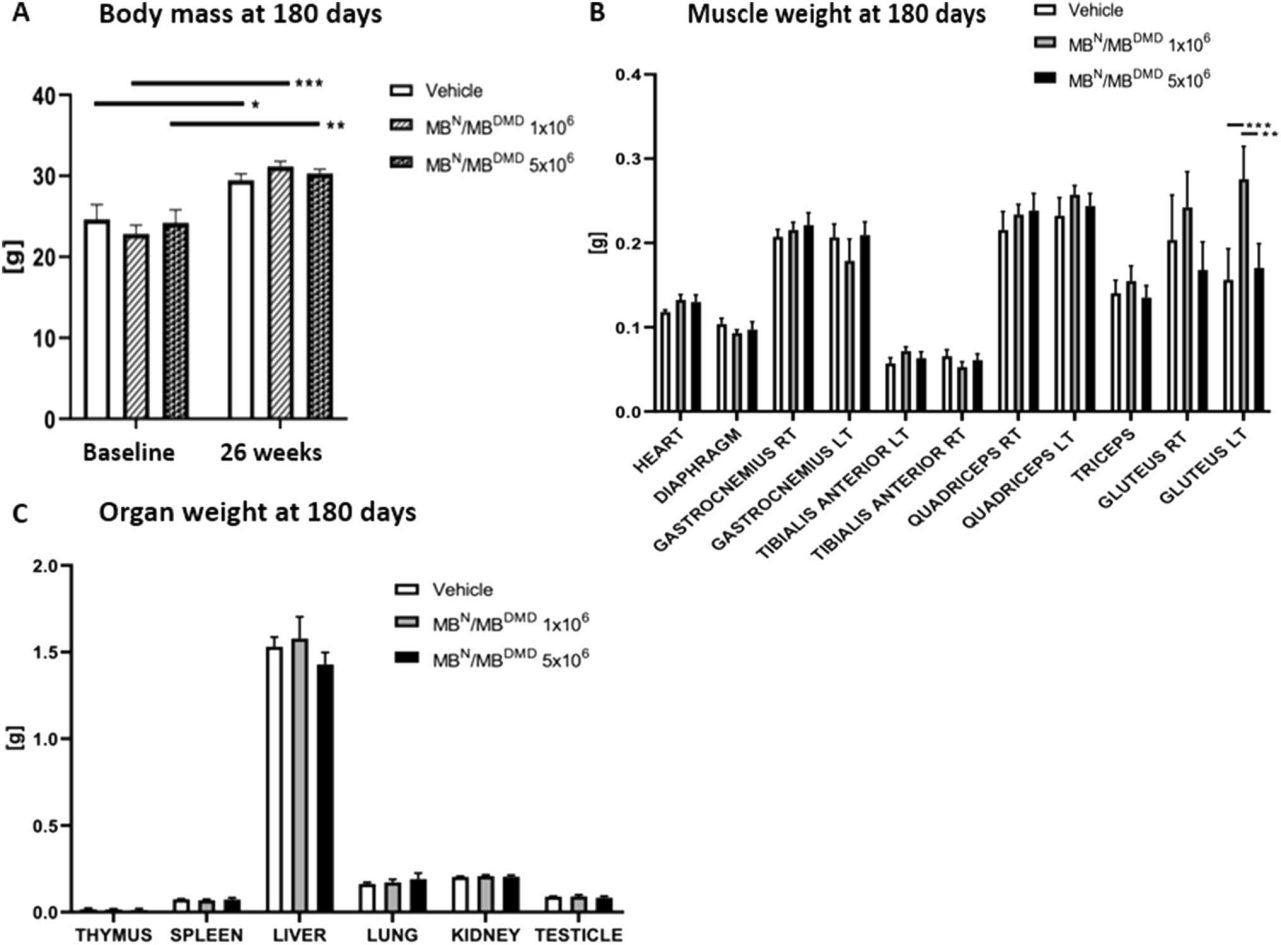
Monitoring of body mass, muscle and organ weights confirms lack of toxicity, and safety of human DEC Therapy at 180 days after intraosseous administration. **A.** Body mass analysis in *mdx/scid* hosts at 180 days after systemic-intraosseous administration of DEC therapy. **B**. Selected muscles weights of the *mdx/scid* mice (heart, diaphragm, gastrocnemius right,  gastrocnemius left,  tibialis anterior, quadriceps right, quadriceps left, triceps and gluteus right and left) at 180 days after systemic-intraosseous administration of DEC therapy (1 × 10^6^ and 5 × 10^6^) compared with the vehicle-injected controls. There were no significant differences between muscle weights of mice injected with DEC when compared with the vehicle-injected controls, further confirming safety and lack of toxicity of DEC therapy. There was no abnormal growth or muscular atrophy observed. **C.** Comparison of the weight of the selected organs (thymus, spleen, liver, lung, kidney and testicle) of the *mdx/scid* mice at the study endpoint of 180 days after systemic-intraosseous administration of DEC therapy (1 × 10^6^ and 5 × 10^6^) revealed lack of abnormalities and weight stability which was comparable with the vehicle-injected controls. There were no significant differences between the organ weights of the mice injected with DEC (1 × 10^6^ and 5 × 10.^6^) and of the vehicle controls. Data presented as mean ± SEM. Two-way ANOVA with Sidak’s multiple comparisons test, *p < 0.05, **p < 0.01, ***p < 0.001

There was no weight loss recorded in *mdx/scid* mice treated with both doses of DEC therapy (1 × 10^6^ and 5 × 10^6^) as well as in the control vehicle-injected mice, over the long-term 180 day follow-up period. At the study baseline, the vehicle-injected mice weighed, on average, 24.66 ± 1.80 g and the body weight increased to 29.47 ± 0.77 g at the 180 day study endpoint. The baseline average weights for 1 × 10^6^ and 5 × 10^6^ DEC treatment groups were 22.84 ± 1.08 g and 24.20 ± 1.63 g respectively and increased to 31.18 ± 0.61 g and 30.33 ± 0.47 g, respectively, at the 180 day study endpoint. Overall, a normal physiological increase in the body weight was observed in both DEC injected and the vehicle-injected animals at 180 days after systemic-intraosseous administration of human DEC (Fig. 3A). Muscle weight analysis after 180 days, confirmed lack of significant differences between the vehicle-injected controls and both DEC treatment groups (1 × 10^6^ and 5 × 10^6^), except for left gluteus muscle, showing increased weight (0.276 ± 0.039 g) when compared with vehicle-injected controls (0.156 ± 0.037 g (Fig. 3B). At the 180 day study endpoint, maintenance of organ weight was confirmed for *mdx/scid* mice treated with both doses of DEC therapy (1 × 10^6^ and 5 × 10^6^) and was comparable with the vehicle-injected controls (Fig. 3C).

### Presence of Human DEC Cells in Target Organs of Heart, Diaphragm and GM was Confirmed by Human HLA-ABC Antigen Expression at 180 Days After Systemic-Intraosseous DEC Administration

**Fig. 4:**
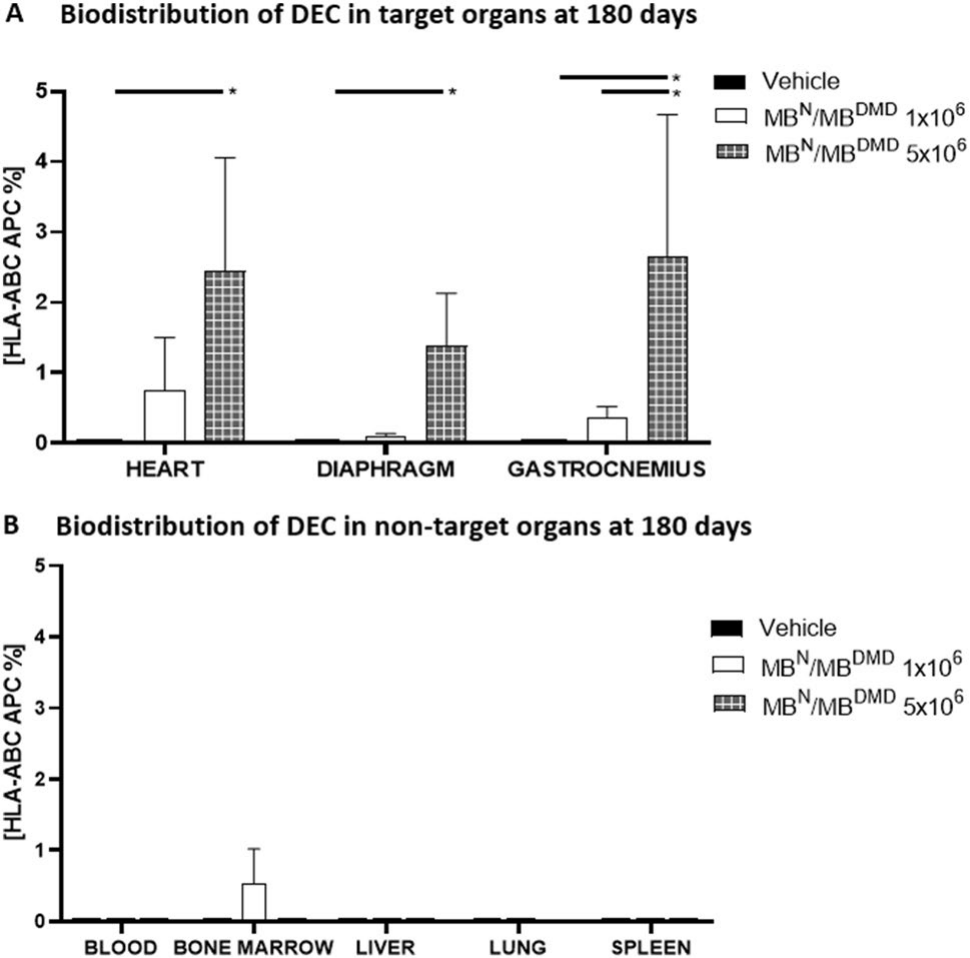
Assessment of the expression of human HLA-ABC (human leukocyte antigen-ABC) confirm biodistribution of human DEC (1 × 10^6^) and (5 × 10^6^) therapy to the A. target and B. non-target organs at 180 days after intraosseous administration to the *mdx/scid* mice. **A.** Dose dependent effect of HLA-ABC antigen expression in the target organs of heart, diaphragm and gastrocnemius muscle at 180 days after administration of two doses of DEC (1 × 10^6^ and 5 × 10^6^) therapy to the m*dx/scid* compared to vehicle-injected controls where a lack of HLA-ABC antigen expression was observed. **B.** Negligible expression of human HLA-ABC antigen after administration of human DEC (1 × 10^6^ and 5 × 10.^6^) therapy to the non-target organs of blood, bone marrow, liver, lungs, and spleen of *mdx/scid* mice compared to the vehicle-injected controls. Data presented as mean ± SEM. Two-tailed Student *t*-test for group comparisons was used to define statistical significance; *p < 0.05

Assessment of biodistribution of human DEC therapy by detection of human HLA-ABC antigen, confirmed presence of cells of the human origin in the target organs of heart, diaphragm and GM at 180 days after systemic-intraosseous administration of both DEC therapy doses (1 × 10^6^ and 5 × 10^6^ cells). In contrast, there were no cells of human origin in the vehicle-injected controls. Moreover, the presence of HLA-ABC positive cells in the heart, diaphragm and gastrocnemius revealed a dose-dependent effect (Fig. 4A). These findings confirm trafficking of human DEC cells from the bone marrow compartment—the site of DEC therapy injection, leading to engraftment and persistence of DEC in the DMD affected muscle tissues, up to 180 days after systemic DEC administration.

### Safety of Human DEC Therapy was Confirmed by Negligible Presence of HLA-ABC Antigen Expression in the Non-Target Organs at 180 days After Intraosseous DEC Administration

DEC therapy safety was assessed at the 180 day study endpoint by detection of DEC trafficking and biodistribution to the non-target organs, by assessment of the human HLA-ABC marker after systemic administration of both DEC therapy doses of 1 × 10^6^ and 5 × 10^6^ cells. The data analysis of expression of human HLA-ABC antigen performed by flow cytometry demonstrated negligible presence of the human HLA-ABC marker in the peripheral blood and bone marrow compartment as well as in the non-target organs including liver, lungs and spleen (Fig. 4B). These findings were comparable with the vehicle-injected controls. This data proves the lack of migration of DEC cells to the non-target organs further confirming the long-term safety of human DEC therapy at 180 days after systemic-intraosseous administration.

### Confirmation of Human Origin of Dystrophin in Cardiac, Respiratory and Skeletal Muscles by Co-localization of Dystrophin with Human Spectrin at 180 Days After Systemic-Intraosseous Administration of Human DEC Therapy

**Fig. 5:**
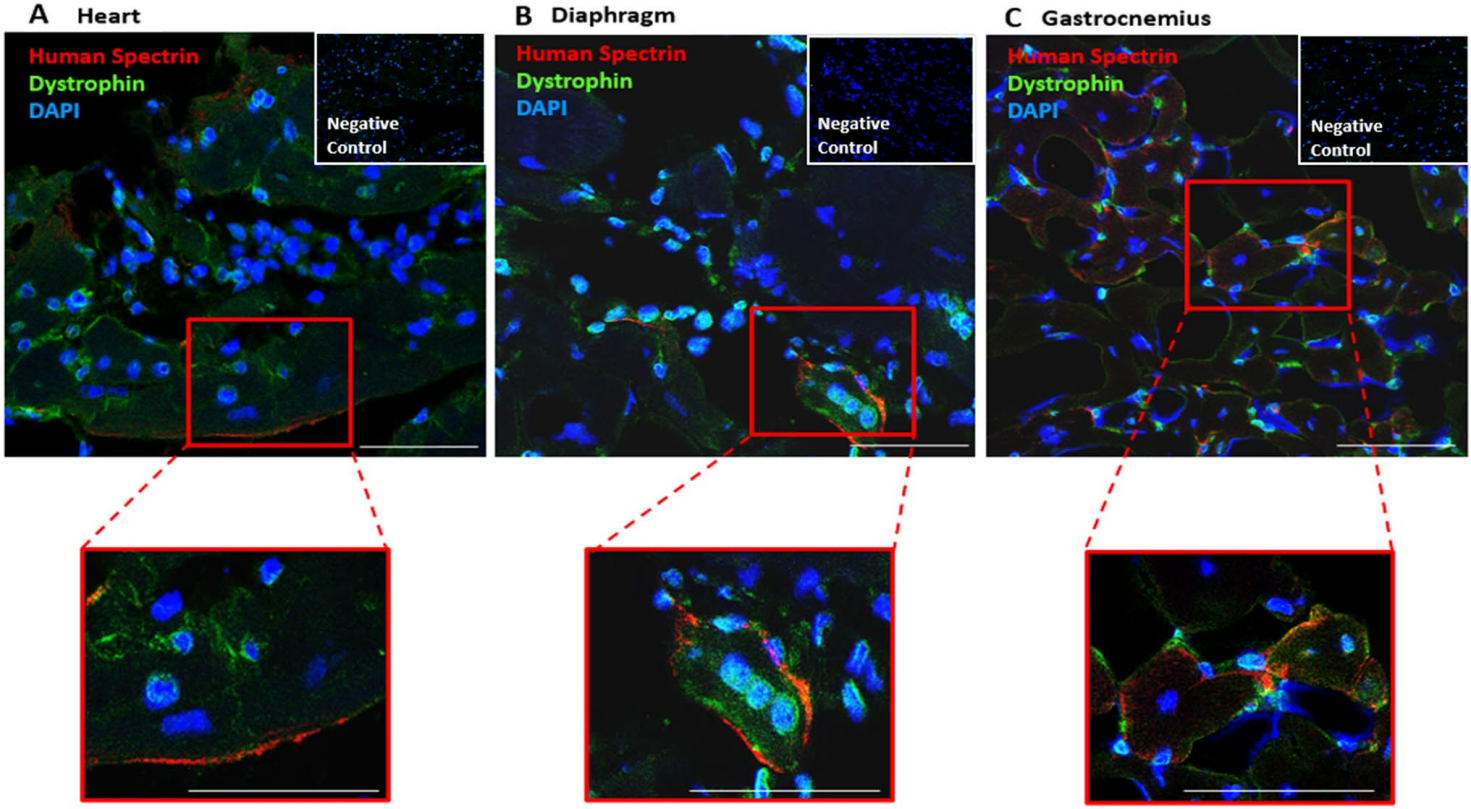
Immunofluorescence analysis of co-localization of dystrophin with human spectirin confirms biodistribution of DEC to target organs and human origin of dystrophin assessed at 180 days after intraosseous administration of DEC (5 × 10^6^) therapy to the *mdx/scid* mice. Representative immunofluorescence images confirming expression and co-localization of dystrophin (green) with human spectrin (red) assessed in the target organs of: **A.** heart, **B.** diaphragm and **C.** gastrocnemius muscle of the *mdx/scid* hosts injected with human DEC (5 × 10.^6^) therapy. Nuclei counterstained with DAPI (blue); Magnification of upper panel 20X, scale bar 20 μm, Magnification of the lower panel 140X, Negative controls are presented on right, upper corner with magnification 20X (ZEISS 710 META, Oberkochen, Germany). Figure adapted from the author’s publication at the Stem Cell Rev Rep journal (Siemionow et al. 2022)

We previously reported restoration of dystrophin expression after systemic-intraosseous administration of human DEC cells to the *mdx/scid* mice (Siemionow et al. 2018b, 2021a, 2022). In the current study, human origin of dystrophin after DEC therapy administration was confirmed by co-localization of dystrophin with the human spectrin in the selected target organs of heart (Fig. 5A), diaphragm (Fig. 5B) and GM (Fig. 5C) after administration of 5 × 10^6^ DEC cells. Co-localization of human spectrin with dystrophin was confirmed at the intracellular side of the membrane. Confirmation of human origin of dystrophin in the target organs after systemic DEC administration further confirms selective migration of DEC cells from bone-marrow compartment to the target organs, further confirming long-term safety and efficacy of DEC therapy at 180 days after systemic-intraosseous administration.

## Discussion

DMD is a progressive and lethal genetic disease caused by mutations of the dystrophin gene (Crisafulli et al. 2020). Lack or malfunctioning of dystrophin, a protein essential for muscle fiber integrity, results in severe muscle degeneration, wasting, and weakness (Falzarano et al. 2015) and leads to premature death due to cardiopulmonary failure. At present, different supportive measures including corticosteroids, ACE-inhibitors, beta blockers and respiratory support are aiming to extend life expectancy of DMD patients; however, these supportive measures do not have an effect on the underlying cause of the disease (McNally et al. 2015).

Currently, there is no cure for DMD. Several potential gene therapies aiming to restore dystrophin including: exon skipping, gene editing, gene splicing and microdystrophin gene delivery via AAVs were tested in both the preclinical studies and clinical trials, however their efficacy in restoration of dystrophin in target tissues has been limited (Dumont et al. 2015). Furthermore, the risk for the development of the adverse immune responses with the AAV-based therapies and the potential for off-target mutations and tumorigenicity related to gene therapies raises significant safety concerns (Gait et al. 2019; Kornegay et al. 2010; Sun et al. 2020). Moreover, repeated dosing of gene therapies may be limited due to sensitization (Doetschman and Georgieva 2017).

Due to these unfavorable side effects found in the historical development of DMD gene therapies, cell-based therapies of myoblast origin have been tested as an alternative option for DMD (Gussoni et al. 1997; Skuk and Tremblay 2015, 2016). Satellite cells have also been considered as an attractive option for cell therapies, due to their self-renewal abilities similar to stem cells. However, assessment of safety and efficacy in the clinic revealed limited migration to muscle tissues, and accumulation in small vessels leading to thrombogenic effects (Sun et al. 2020). Other approaches including application of mesoangioblasts, cardiomyocytes, allogeneic cardiosphere-derived cells and MSC in experimental models and clinical trials are encouraging, but due to different efficacy results of donor cell engraftment and lack of significance in functional improvements represents major limitation of these therapeutic approaches (Barthélémy and Wein 2018; Biressi et al. 2020; Cossu et al. 2015; Davis 2022; Rajput et al. 2015; Skuk and Tremblay 2015). Recent reports on potential therapeutic effects of the induced pluripotent stem cells (iPS) in muscular dystrophies revealed a major challenge in achieving satisfactory differentiation of iPS toward MB lineage (Miura et al. 2022). Therefore limited engraftment, transient functional improvements, low cell survival, and the need for immunosuppression to prevent cell rejection precluded their routine clinical applications.

Considering the safety concerns of gene therapies and efficacy concerns of the stem-cell-based therapies, it is clear that there is a need to develop a safer and more efficacious therapeutic approach for DMD patients. Our DEC therapy has addressed these unmet needs. We have reported both the concept and the rationale for application of chimeric cell therapy in DMD (Siemionow et al. 2018a, 2018b).

We have planned our studies and have chosen the DEC dose based on the recommendations of the World Health Organization (2013) for cell therapies dosing in the experimental models. We have also tested different doses of human DEC therapy based on the conversion of the dose from mice to humans (Elmeliegy et al. 2021; Nair and Jacob 2016) and different routes of DEC cell delivery including intramuscular, intraosseous and intravenous routes (Siemionow et al. 2018a, 2018b, 2019, 2021a, 2021b, 2022, unpublished data). Since the intraosseous route of DEC administration was found to be most efficacious for cell engraftment corresponding with increased dystrophin expression and improvement of function, we have further assessed systemic effects of DEC on cardiac, respiratory and skeletal muscles in both the immunocompetent mdx and immunocompromised *mdx/scid* mouse models (Siemionow et al. 2019, 2021a, 2022). Recently, we have reported long-term efficacy of human DEC therapy assessed at 90 (Siemionow et al. 2021a) and 180 days (Siemionow et al. 2022) following systemic-intraosseous DEC administration. We have performed functional, histomorphological, and immunofluorescent assessments of the crucial for DMD patients’ survival organs of heart, respiratory muscle (diaphragm), and skeletal muscle (gastrocnemius) to confirm the long-term systemic effect of human DEC therapy. One of the major challenges of cell-based therapies for DMD is restoration and long-term maintenance of dystrophin, and we have confirmed dose-dependent functional efficacy of DEC therapy which correlated with increased dystrophin expression (Siemionow et al. 2021a, 2022). Furthermore, we have confirmed significant improvement in the mdx pathology revealed by decreased number of the centrally nucleated fibers, reduced inflammation, and fibrosis in heart, diaphragm and GM which correlated with amelioration of functional decline in the cardiac, respiratory, and skeletal muscles confirmed by standard functional tests of echocardiography, plethysmography, and muscle strength tests, assessed at 90 and 180 days after systemic-intraosseous DEC administration (Siemionow et al. 2021a, 2022).

Thus, to bring DEC therapy closer to clinical application, we used the *mdx/scid* mouse model of DMD to test human cell line created from normal and DMD affected human donors according to the same manufacturing protocol which will be used in the clinical scenario. This is responding to the regulatory requirements indicating that preclinical efficacy studies should be testing the same type of cell-based therapy which will be applied in the clinical trials. Immunocompromised mice allowed us to prevent rejection of the tested human cells by the xenogeneic host’s immune system and provided the best opportunity to monitor DEC cells’ biodistribution, engraftment and to detect any evidence of the tumorigenic potential of the administered cells of human origin.

It should be noted that the use of *mdx/scid* mouse model, limits evaluation of the potential immune response, however we have confirmed in the immunocompetent mdx mouse model that DEC therapy created from two unrelated donors resulted in significant increase in dystrophin expression correlating with improvement of function of skeletal and cardiac muscle after both intramuscular and intraosseous administration without the need for immunosuppression (Siemionow et al. 2018a, 2019).

Therefore, the goal of the current study was to assess the long-term safety of human DEC therapy at 180 days after systemic-intraosseous administration to the *mdx/scid* mouse model. We based our preclinical safety study on the recommendations of the regulatory agencies for a potential therapeutic application of DEC (European Medicine Agency 2008) These assessments included: evaluation of safety of DEC creation, monitoring of the local and systemic side effects, monitoring of the dose-dependent local and systemic toxicity, potential for tumorigenicity, effect of DEC cells administration on animals’ well-being and muscle and organs weight at autopsy, as well as assessment of DEC biodistribution testing human DEC cell trafficking and engraftment to the target and non-target organs of the *mdx/scid* mice recipient.

First, to ensure the long-term safety and lack of genotoxicity of the DEC cells created via ex-vivo cell-fusion, we performed COMET assay at different cell passages of the parent cells and the fused DEC cells and confirmed DNA stability and lack of DNA damage (Siemionow et al. 2021a, 2021b), revealed by the lack of presence of the comet-like tail structures characteristic for DNA damage, thus confirming safety of the created DEC therapy for systemic-intraosseous administration.

Next, we confirmed the lack of local and systemic side effects pertinent to the procedure of intraosseous administration of DEC cells. Moreover, we confirmed lack of the local and/or systemic toxicity in response to the administration of the low (1 × 10^6^) and high dose (5 × 10^6^) of DEC therapy which was well tolerated both during intraosseous administration as well as over the entire study follow-up period of 26 weeks.

Since tumorigenicity is considered one of the most challenging aspects of current gene as well as cell-based therapies, we have assessed animals by MRI scanning before and at 30-, 90- and 180 day study endpoint after DEC therapy administration. The T2 weighted images of brain, chest, and pelvis as well as the T1 weighted images for brain and liver, confirmed the lack of tumor formation over the entire long-term follow-up period.

Moreover, the lack of tumorigenicity confirmed by MRI was also confirmed in all animals except one, with hind limb mass found during routine observation. Further analysis revealed sarcoma of murine origin. The pathology abnormalities and development of spontaneous malignant sarcomas are common in the mdx mice due to their immunocompromised state and have been recorded by other investigators in a tumorigenicity study of *mdx/scid* mice (Santagostino et al. 2017).

To further investigate safety of DEC cell therapy and to ensure the mice tolerated the transplant well, we performed body mass analysis at baseline as well as after 26 weeks (180 days) after intraosseous DEC injection. We observed a normal physiological weight gain throughout the study, both in the DEC injected mice as well as in the vehicle-injected controls. There was no weight loss observed in any animal, which might have suggested illness or poor therapy tolerance. Moreover, during autopsy at the study endpoint, we also weighed muscles and selected organs of both the vehicle-injected controls and DEC treated *mdx* mice to assess for any abnormal weight increase suggestive of tumor formation in organs or weight loss in muscle suggesting muscle atrophy. However, no abnormal changes were observed at long-term follow-up, thus we concluded that intraosseous DEC cell injection was safe in the long-term follow-up and caused no abnormal changes. Furthermore, we have observed that the average weight of muscles after 180 days was generally higher in the DEC therapy groups than vehicle-injected controls indicating the therapeutic effect of the DEC therapy on the skeletal muscles. In addition, body mass, muscle and organ weights assessments during autopsy performed at the 180 day study endpoint, confirmed normal physiological animal growth comparable with the vehicle injected control mice, further confirming lack of deleterious effect of DEC therapy on the animals’ wellbeing over the entire 26 week period post DEC administration.

Furthermore, we have proven that intraosseous DEC transplant was corresponding with long-term engraftment and biodistribution of the human DEC cells from the injection site of the bone marrow compartment to the target organs of heart, diaphragm, and GM as confirmed by the presence of human HLA-ABC marker expression. Moreover, the lack of HLA-ABC marker in the non-target organs of blood, bone marrow, liver, lungs, and spleen confirmed safety of intraosseous DEC administration by the lack of evidence of DEC cell migration and lodging in the lungs. This supports the benefits of the intraosseous when compared to intravenous administration of cell-based therapies (Goto et al. 2018, 2021; Lee et al. 2013; Marktel et al. 2019; Siemionow et al. 2005) where the “first-pass” accumulation of cells in the lungs was reported for MSC delivered via intravenous route (Krueger et al. 2018).

In addition, the presence of DEC cells of human origin in the target organs of heart, diaphragm, and GM, was demonstrated by co-localization of dystrophin expression with the human spectrin staining. These findings further confirmed trafficking, long-term engraftment and efficacy of human DEC cells which correlated with amelioration of cardiac, pulmonary and skeletal muscle function confirmed by echocardiography, plethysmography and standard muscle strength tests respectively as reported in our previous study (Siemionow et al. 2022).

It is important to address the limitations of the current study, including testing of DEC therapy in the immunocompromised *mdx/scid* mouse model, which allows assessment of human cells trafficking and engraftment to the target and non-target organs, but may mask or limit the potential immune response. However, we have addressed these limitations by testing DEC therapy in the immunocompetent mdx mouse model where efficient cell engraftment correlated with dystrophin expression and significant improvement of function confirmed after both, the local (Siemionow et al. 2018a, 2018b) as well as systemic-intraosseous administration of DEC without any evidence of side effects and without the need for immunosuppressive therapy (Siemionow et al. 2021a, 2022).

Advantage of using the *mdx/scid* mouse model of DMD includes ability to test human DEC cells created from normal and DMD affected donors according to the same manufacturing protocol which will be used in the clinical scenario, and as such fulfills the regulatory requirements for the preclinical safety and efficacy studies for a new, cell-based ATMP therapies which will be applied in the future clinical trials (Siemionow et al. 2021a, 2022). The same approach of using immunocompromised NOD-*scid* and NSG mice models was reported in the preclinical study validating human skin-derived ABCB5-positive mesenchymal stromal cells for clinical application (Tappenbeck et al. 2019). These authors emphasized the importance of testing ATMP therapy of human cell origin in the immunocompromised animal models, to validate the new therapy manufactured according to the clinically relevant protocol (ClinicalTrials.gov: https://clinicaltrials.gov/ct2/show/NCT04971161). However, there are some limitations of immunocompromised mouse models, including the risk and susceptibility of developing the spontaneous tumor formations as observed in one mouse in our study and also reported by other investigators (Santagostino et al. 2017; Tappenbeck et al. 2019).

Furthermore, in contrast to gene therapies targeting the specific dystrophin mutations, thus limiting their clinical applications to the restricted number of patients, our DEC therapy does not require genetic manipulation or application of viral vectors and as such is potentially safer and applicable to all muscular dystrophies regardless of gene mutation including the limb-girdle muscular dystrophies and others.

In summary, long-term engraftment and biodistribution of DEC cells to the selected target organs of heart, diaphragm and GM was confirmed by presence of the human HLA-ABC antigen expression in the respective target organs at 180 days after systemic-intraosseous DEC administration. Moreover, there was negligible presence of human HLA-ABC markers in the non-target organs of lung, liver, and spleen, confirming safety of DEC therapy. In addition, we confirmed co-localization of dystrophin with human spectrin, in the heart, diaphragm and GM, further confirming migration of human DEC cells from the injection-site of bone marrow compartment into the target organs as well as human origin of the dystrophin at 180 days after systemic intraosseous DEC administration.

## Conclusions

In this study, we assessed long-term safety of DEC fusion protocol after systemic-intraosseous administration to the *mdx/scid* mouse model. We confirmed lack of DNA damage before and after cell fusion procedure by COMET assay, confirming lack of genotoxicity and safety of human DEC therapy. Moreover, lack of abnormal growth and tumor formation was confirmed by weekly animal palpation and by MRI scanning of selected organs before and after DEC administration as well as at the study endpoint by autopsy. Assessment of body weight and selected muscle and organ weights revealed normal physiological growth of animals over the entire 26-week follow-up which was comparable with the assessments of the vehicle injected controls. Biodistribution of DEC to target and non-target organs assessed by expression of HLA-ABC antibody confirmed human DEC trafficking to the heart, diaphragm and GM of the *mdx/scid* host. Moreover, the negligible presence of DEC in the non-target organs further confirmed DEC therapy safety. Further assessment of biodistribution of DEC to the selected target organs after systemic administration was confirmed by co-localization of dystrophin with human spectrin at 26 weeks after administration. The long-term assessments of safety, toxicity and biodistribution performed in this study confirmed, both the safety as well as selectivity of DEC therapy in targeting the DMD- affected organs. Moreover, these findings correlated with lack of tumorigenicity, confirmed by MRI and preservation of normal physiological growth and wellbeing of the DEC-injected animals. Thus, to the best of our knowledge this is the first report on the long-term safety of DEC therapy for DMD. Our preclinical safety and tolerability data represent a valuable contribution towards the unmet medical needs and introduces DEC as a novel, efficient and safe therapy for Duchenne muscular dystrophy patients.

## Data Availability

All data generated in this study are presented in the manuscript and are available for presentation upon request.

## Abbreviations

AAV: Adeno-associated virus
ATMP: Advanced therapy medicinal product
CD: Cluster of differentiation,
COMET assay: Single-cell gel electrophoresis
DAPI: 4′,6-Diamidino-2-phenylindole
DEC: Dystrophin expressing chimeric
DMD: Duchenne muscular dystrophy
DMSO: Dimethyl sulfoxide
EDTA: Ethylenediaminetetraacetic acid
FACS: Fluorescence-activated cells sorting
FBS: Fetal bovine serum,
GM: Gastrocnemius muscle
HEPES: 4-(2-Hydroxyethyl)-1-piperazineethanesulfonic acid
HLA: Human leukocyte antigen
iPS: Induced pluripotent stem cells
MB: Myoblast
Mdx: X chromosome-linked muscular dystrophy
MSCs: Mesenchymal stem cells
MRI: Magnetic resonance imaging
PBS: Phosphate-buffered saline
PEG: Polyethylene glycol
